# Psychometric Properties of the Healthy Lifestyle Behaviors Scale in Portuguese Pregnant Women

**DOI:** 10.1177/01939459241245217

**Published:** 2024-04-14

**Authors:** Maria Arminda Nunes, Bernadette Mazurek Melnyk, Sofia Almeida, Alexandrina Cardoso, Margarida Vieira

**Affiliations:** 1 Centre for Interdisciplinary Research in Health, Institute of Health Sciences, Universidade Católica Portuguesa, Porto, Portugal; 2 College of Nursing, The Ohio State University, Helene Fuld Health Trust National Institute for Evidence-based Practice in Nursing and Healthcare, Columbus, OH, USA; 3 Nursing School of Porto, Porto, Portugal

**Keywords:** healthy lifestyle, behaviors, pregnant women, outcomes, psychometrics

## Abstract

**Background:**

Pregnancy provides a privileged and opportune moment to implement interventions promoting healthy lifestyle behaviors and significantly improving perinatal outcomes. The Healthy Lifestyle Behaviors Scale (HLBES) can be used to assess health promoting behaviors, such as diet, physical activity, and mental health.

**Purpose:**

This study aimed to examine the psychometric properties of the HLBES in Portuguese pregnant women.

**Methods:**

A methodological study was conducted on a convenience sample of 192 pregnant women receiving prenatal care. After cross-cultural adaptation, an exploratory factor analysis and internal consistency assessment were carried out to evaluate the psychometric properties of the scale. Data collected included the Healthy Lifestyle Beliefs Scale to assess the HLBES’ criterion validity.

**Results:**

Exploratory factor analysis with Varimax rotation yielded 2 subscales that explained 45.23% of the total variance. The scale revealed an overall internal consistency of 0.78 and a good criterion validity with the Healthy Lifestyle Beliefs Scale (*r* = 0.65, *P* < .01).

**Conclusion:**

Our results suggest that the HLBES is an instrument for reporting healthy lifestyle behaviors in Portuguese pregnant women; however, further studies are recommended. This scale can be used to not only describe healthy lifestyle behaviors in pregnant women but also to determine the effects of health promoting interventions.

The adoption of healthy lifestyle behaviors during pregnancy is essential to promote a healthy pregnancy and prevent maternal and fetal complications. Thus, maternal lifestyle behaviors during pregnancy have a significant impact on perinatal outcomes. In addition, pregnancy is a privileged and opportune moment to enhance healthy lifestyle behaviors, as women are more receptive to making positive changes to their lifestyles.^1,2^

The World Health Organization (WHO) defines healthy lifestyle as the behaviors shaped by an individual during the socialization process, include a range of behaviors that prevent preventable disease and promote physical and mental well-being.^3^ Therefore, identifying the healthy lifestyle behaviors such as regular physical activity, balanced diet, adequate sleep, and managing stress^4^ is essential to optimize maternal and infant health during this period.

Physical activity during pregnancy has been associated with a reduced risk of gestational diabetes, preeclampsia, and cesarean section, and contributes to improving the emotional well-being of the pregnant woman.^5-7^ Sedentary behaviors such as increased screen time is associated with overweight, obesity, hypertension, and type 2 diabetes during pregnancy.^8,9^ Similarly, eating a balanced diet is associated with a lower risk of gestational diabetes, preterm delivery, and fetal growth restriction.^10-13^ In addition, maternal diet during pregnancy affects maternal and child health through epigenetic mechanisms.^14^ Physical inactivity and unhealthy eating habits are associated with excessive weight gain and obesity during pregnancy.^15^ Goldstein et al (2018) noticed that 42% of pregnant women in the United States, 30% in Europe, and 10% in Asia were classified as overweight or obese.^16^

Adequate sleep duration and quality is also crucial in reducing the risk of gestational hypertension, preeclampsia, preterm birth, and fetal macrosomia postpartum depression.^17-19^ In addition, there is a significant relationship bet-ween sleep disturbances during pregnancy and postpartum depression,^20^ which highlights the importance of early diagnosis and prenatal intervention. On the other hand, high levels of stress during pregnancy have been associated with adverse outcomes such as low birth weight, preterm birth, and emotional, behavioral, and cognitive issues in the child.^21,22^ In addition, prenatal anxiety and depression have been associated with poor nutrition, excessive weight gain, substance abuse, alcohol, and smoking.^23-25^ Therefore, be-havioral interventions aimed at reducing stress can improve healthy lifestyle behaviors.^24^

In conclusion, several adverse perinatal outcomes are associated with lifestyle behaviors. It is therefore important to develop and implement interventions to promote healthy lifestyle behaviors in pregnant women, which can contribute to improving maternal and infant health, reducing the risk of complications, and contributing to a healthier start in life. Identifying pregnant women who have unhealthy lifestyle behaviors can be useful for developing preventive interventions.

In this context, the Healthy Lifestyle Behaviors Scale (HLBES) can help identify high-risk women and monitor the efficacy of health promoting interventions. The HLBES was developed by Melnyk to assess changes in healthy lifestyle behaviors related to diet, physical activity, and mental health,^26^ and has been used in studies in the United States on populations of adolescents,^26-28^ parents with preschool children,^29^ students,^30^ and academic and nonacademic staff in university settings^31^ with Cronbach alphas ranging from 0.78 to 0.86.

## Purpose

This study reports on a methodological cross-cultural adaptation (phase I) and analysis of the psychometric properties of the Portuguese version of the HLBES (phase II). The main purpose of the study was to examine the psychometric properties of the HLBES in Portuguese pregnant women.

## Methods

### Phase I: Cross-cultural Adaptation

The recommendations in the study by Beaton et al,^32^ were followed in the cross-cultural adaptation procedure of the HLBES in 5 steps: (1) Portuguese translation by 2 independent bilingual experts; (2) the synthesis of the translations was made after the analysis of the 2 translated versions by the research team and the bilingual experts; (3) back translation was conducted by 2 certified native English-speaking translators, confirming the linguistic equivalence to the original scale; (4) the first version was revised by a committee of experts, confirming its content validity; (5) the pretest was administered to 18 pregnant women receiving prenatal care.

### Phase II: Psychometric Properties

#### Participants

A convenience sample of 192 pregnant women receiving prenatal care in public primary care clinics was recruited. This study was approved by the Ethics Committee of ARS-Norte (protocol CE/2022/99) after authorization from the original author of the scale. All pregnant women signed an informed consent form. The data collected were processed to maintain the anonymity and confidentiality of the participants.

#### Measures

In the study, sociodemographic questions were included along with the HLBES, such as age, nationality, gestational age, and level of education completed.

##### Healthy lifestyle behaviors scale

The HLBES is a 15-item, self-report Likert-type scale developed to measure healthy lifestyle behaviors related to physical activity, diet, and mental health. The answers range from 1 (strongly disagree) to 5 (strongly agree), and the total possible score can vary from 15 to 75. Examples of items include “I eat 5 servings of fruits and vegetables daily,” “I engage in muscle-strengthening activities on 2 or more days of the week,” and “I set goals I can accomplish.” In a pilot study with pregnant women, the Cronbach’s alpha was 0.86.^33^

##### Healthy lifestyle beliefs scale

In this study, we utilized the Portuguese version of the Healthy Lifestyle Beliefs Scale to assess the HLBES’ criterion validity, since studies highlight positive correlations between them.^27,29,34^ The Healthy Lifestyle Beliefs Scale assesses individuals’ beliefs regarding their capacity to maintain a healthy lifestyle.^35^ This scale is a self-report instrument consisting of 15 items scored on a Likert-type scale ranging from 1 (strongly disagree) to 5 (strongly agree). Some of the items on the scale include “I am sure that I will feel better about myself if I exercise regularly,” “I know that I can make healthy snack choices regularly,” and “I am sure that I can handle my problems well.” The total score on the Healthy Lifestyle Beliefs Scale ranges from 15 to 75, and the Portuguese version has demonstrated good internal consistency (α = 0.83) among pregnant women.^36^

### Statistical Analysis

The recommendations of DeVellis and Thorpe (2022) were followed for statistical analysis.^37^ SPSS Statistics version 28.0 software was used to enter and analyze the data. De-scriptive statistics were computed to summarize sociodemographic characteristics and the scale items. Mean and standard deviation (SD) were used to describe continuous variables, while absolute and relative frequencies were used for categorical variables.

The validity of the construct was examined through exploratory factor analysis (EFA) with principal component analysis (varimax rotation), using initial eigenvalues >1 to determine the number of components, with factor loadings >0.4 considered significant.^37^ The appropriateness of the data for EFA was assessed using the Kaiser-Meyer-Olkin (KMO) test (≥0.8) and Bartlett’s test (significant at <0.05). Before the EFA, we analyzed the correlation matrix showing the relationships between the HLBES items, considering a minimum correlation coefficient of 0.30.

To estimate the reliability of the internal consistency of the HLBES and each identified subscale, the Cronbach’s alpha coefficient, the corrected item-total correlation, and the alpha estimated after deletion of an item were used. Based on the relationship between HLBES and the Healthy Lifestyle Beliefs Scale, the Pearson correlation coefficient between the target scale and the Healthy Lifestyle Beliefs Scale was calculated to assess its concurrent validity. The higher the correlation coefficient is, the higher the concurrent validity. We analyzed the correlations between the HLBES, its subscales, and sociodemographic variables.

## Results

### Phase I: Cross-cultural Adaptation

During the synthesis phase, we analyzed both translations and discovered minimal differences, mainly related to grammatical structure and words with similar meanings.

The Expert Committee was composed of a nurse-midwife with more than 10 years of experience, a maternal health and midwifery researcher, and a nursing researcher with methodological experience. Cross-cultural adjustments were made to 4 items to ensure semantic and experiential equivalence to the Portuguese context, in particular items 4, 7, 8, and 10.

Item 4 “I limit television viewing and video game playing to two hours per day or less” was modified to “I limit screen time (TV, cell phone, video games) to two hours per day or less.” This item refers to screen time, regardless of the device used, which has a significant impact on physical and mental health. Excessive screen time is associated with several negative health outcomes, including increased sedentary behavior, sleep disorder, and negative mental health outcomes such as increased stress and anxiety.^38-40^ Since Portuguese aged 16 to 64 years spend screen time on smartphones extensively, in addition to television and video games,^41^ we decided to include them to capture this behavior among Portuguese pregnant women.

Considering the WHO guidelines to limit sugary drink consumption,^15,42^ we have adjusted items 7 and 8 regarding sugar-sweetened beverage intake. Item 7 “I drink only two sugared drinks a day (for example, regular soda or juice)” was replaced by “I do not drink sugared drinks (soda or juice).” Similarly, item 8 “I choose water as a beverage instead of a sugared drink at least once per day” was substituted by “I choose water as a beverage instead of a sugared drink.” These adaptations were made in recognition of the adverse effects on perinatal outcomes associated with excessive sugar consumption during pregnancy.^43^ Items 7 and 8 retain the same significance as the original scale, but without implying that sugary drinks should be allowed during pregnancy, thus aligning the scale items with evidence-based recommendations for promoting healthy behaviors during pregnancy.

The adjustment made to item 10 from “I eat at least three meals a week with my friends” to “I speak at least three times a week with my friends” aims to capture reports of social support from pregnant women. Both sharing meals and talking to friends can contribute to increasing social support.^44^ By modifying the item to prioritize talking to friends rather than specifically eating together, we aim to capture the fundamental aspects of social interaction and support, while recognizing the necessary adaptability during pregnancy. During this period, various factors, including limitations on physical activity due to obstetric conditions, time limitations, and financial constraints, can make it difficult to meet up for meals. In addition, data from 2023 shows that the Portuguese eat 71.5% of their meals at home compared to 28.5% outside the home.^45^

During the pretest, we conducted interviews with pregnant women who had completed the scale. The interviews revealed that the women had a clear understanding of all the items, and as a result, no significant changes were made to the scale. The final version was reported back to the author of the scale, who approved the adaptations.

### Phase II: Psychometric Properties

#### Sample characteristics

A total of 192 pregnant women gave informed consent and completed the questionnaire. The mean age of the participants was 32.2 years (SD = 4.9) and ranged from 18 to 46 years. One hundred eighty-five (96.4%) of the participants had Portuguese nationality. The mean gestational age of the participants was 26.1 weeks (SD = 7.97) and ranged from 8 to 39 weeks. In this study, 103 (53.6%) participants had a high school education, 71 (37.0%) had a 12th grade, 15 (7.8%) had a 9th grade, and 3 (1.6%) had a 6th grade education.

#### Exploratory factor analysis

The mean values of the scale items ranged from 2.68 (SD = 1.22) for item 3 “I engage in muscle-strengthening activities on two or more days of the week” to 4.33 (SD = 0.75) for item 8 “I choose water as a beverage instead of a sugared drink” (Table 1). Item 2 relative to physical activity has a mean score like item 3.

**Table 1.** HLBES Items With Principal Components Factor Loadings and Reliability Results (N = 192).

Item	Mean (SD)	Component factor loadings		Corrected item-total correlation	Cronbach’s alpha if item deleted
		1	2		
Item 1: I eat 5 servings of fruits and vegetables daily	3.61 (1.06)		**0.53**	0.36	0.78
Item 2: I engage in aerobic physical activity of at least moderate intensity for at least 150 min/week. or 75 min/week of vigorous intensity	2.91 (1.19)		**0.85**	0.50	0.76
Item 3: I engage in muscle-strengthening activities on 2 or more days of the week	2.68 (1.22)		**0.80**	0.44	0.77
Item 4: I limit screen time (TV, cell phone, video games) to 2 hours per day or less	3.03 (1.15)	—	—		
Item 5: I say something positive to my family member or friends every day	4.15 (0.74)	**0.54**		0.21	0.79
Item 6: I eat low-fat foods in my diet	3.83 (0.78)	**0.57**		0.54	0.76
Item 7: I do not drink sugared drinks (soda or juice)	3.63 (1.07)	**0.50**		0.41	0.77
Item 8: I choose water as a beverage instead of a sugared drink	4.33 (0.75)	**0.66**		0.51	0.76
Item 9: I set goals I can accomplish	3.93 (0.81)	**0.56**		0.46	0.77
Item 10: I speak at least 3 times a week with my friends	4.25 (0.85)	**0.67**		0.23	0.79
Item 11: I do not add salt to my foods	3.54 (1.34)	—	—		
Item 12: I get a minimum of 7 hours of sleep on most nights	3.86 (1.09)	—	—		
Item 13: I talk about may worries or stress to my friends	3.83 (0.88)	**0.49**		0.37	0.77
Item 14: I do what I should to lead a healthy life	3.71 (0.81)		**0.62**	0.64	0.75
Item 15: I do healthy things to cope with my worries/stress	3.70 (0.77)		**0.49**	0.53	0.76
Variance (%)		31.83	13.40		

Abbreviations: HLBES: Healthy Lifestyle Behaviors Scale; SD: standard deviation.

Preliminary statistical analyses were performed to support the principal components analysis. The Bartlett test (χ^2^ = 669.0; *P* < .001) and the KMO test (0.8) indicate that the quality of correlations between the variables is moderate and the sample size is sufficient for EFA. The correlation matrix between items of the scale showed numerous correlations of 0.30 or greater, except items 4, 11, and 12 had low correlations between all the variables.

The varimax rotation identified 5 components with eigenvalues >1, which were extracted with loading >0.4, explaining 60.4% of the total variance. However, when analyzing internal consistency, component 5, which comprises items 4 and 11, exhibited a Cronbach’s α of less than 0.6.

Successive EFA were conducted from the beginning, after each item had been eliminated, until all components had internal consistency ≥0.7 and factor loading greater than 0.4 for the items. Thus, EFA was repeated fixing the extraction of 4 components. This solution explained 53.1% of the total variance, but the fourth component, which comprises items 4 and 11, had poor internal consistency. A new EFA was performed establishing the extraction of 3 components, which explained 45.6% of the total variance; however, the third component (items 10, 12, and 13) had an α = 0.48. Hence, successive analyses were performed up to a final solution was established with the extraction of 2 components.

However, items 4, 11, and 12 showed a loading factor lower than 0.4 (0.26, 0.21, and 0.24, respectively) so they were excluded in the following analysis. The final factorial solution scale, with items 4, 11, and 12 deleted, allowed the selection of 12 items distributed by 2 components explaining 45.23% of the total variance (Table 1). Subscale 1 contains 7 items and subscale 2 includes 5 items. The items related to diet, physical activity, and mental health were distributed across both subscales, with no cross-loading; all items were saturated in a single component.

#### Criterion validity

The results showed a Pearson correlation coefficient between the total scores of the HLBES and the total scores of the Healthy Lifestyle Beliefs Scale (*r* = 0.65, *P* < .01). The physical activity and social support subscale of the Healthy Lifestyle Beliefs Scale showed a positive, moderate, and statistically significant correlation with the HLBES total score (*r* = 0.48, *P* < .01). The eating habits and screen time subscale of the Healthy Lifestyle Beliefs Scale shows a positive, moderate, and statistically significant correlation with the HLBES total score (*r* = 0.56, *P* < .01). Finally, the coping and problem-solving subscale of the Healthy Lifestyle Beliefs Scale showed a positive, moderate, and statistically significant correlation with the HLBES total score (*r* = 0.47, *P* < .01).

No significant differences were found in the total of HLBES subscale scores according to nationality, gestational age, and cohabiting with the baby’s father. A weak, positive association was observed between the total HLBES score and age (*r* = 0.15, *P* < .05) and between component 1 (*r* = 0.15, *P* < .05) and age and educational level (*r* = 0.15, *P* < .05).

#### Reliability

Cronbach’s alpha for the total scale was 0.78, and the coefficients for the subscales ranged from 0.70 to 0.73. The corrected item-total correlations were greater than 0.30, except for items 5 and 10 (Table 2). However, with the deletion of these items the Cronbach’s alpha would increase by 0.01. The total mean of the Portuguese version of HLBES was 44.64 (SD = 6.03). The mean of subscale 1 was 28.01 (SD = 3.52) and the mean for subscale 2 was 16.61 (SD = 3.60).

**Table 2.** Statistics and Cronbach’s Alpha of HLBES Components (N = 192).

Component	Number of items	Mean (SD)	Range	Cronbach’s alpha
1	7	28.01 (3.52)	7-35	0.70
2	5	16.61 (3.60)	5-25	0.73
Total scale	12	44.64 (6.03)	12-60	0.78

Abbreviations: HLBES: Healthy Lifestyle Behaviors Scale; SD: standard deviation.

## Discussion

Cross-cultural adaptation and validation of instruments allow data collected from different populations, countries, and languages, enhancing the validity and generalizability of the studies carried out.^46^ The HLBES was designed to assess individuals’ healthy lifestyle behaviors in relation to diet, physical activity, and mental health. In this study, we cross-culturally adapted and validated the 15-item HLBES, originating the 12-item Portuguese version of the HLBES.

Based on the psychometric properties of this study, it appears that the HLBES is valid and reliable for measuring healthy lifestyle behaviors among pregnant women in Portugal. However, we suggest further studies are needed to fully understand the associations found in the Portuguese version. The Cronbach’s alpha value of the total scale and the subscales indicates that the Portuguese version of the HLBES has a good reliability (α = 0.78), although slightly lower than other studies that used the scale with the pregnant women,^33^ but similar to that in other studies.^30^

After EFA, 3 items were deleted and a 12-item scale was tested. Item 4 “I limit screen time (TV, cell phone, video games) to two hours per day or less” was excluded. Pregnant women may view screen time differently than they do other healthy lifestyle behaviors such as diet, physical activity, and mental health. In addition, limiting screen time may not be relevant or practical for Portuguese pregnant women, especially due to work reasons. It is important to note that 53.6% of participants had a high school education. During the cross-cultural adaptation process, this item was modified, but during the pretest, pregnant women demonstrated a good understanding of what was meant.

In addition, item 11 “I do not add salt to my foods” was deleted. Pregnant women may prioritize consuming essential nutrients for fetal development over reducing salt intake, despite existing WHO guidelines.^47^ Given that sleep is often compromised during pregnancy,^18^ it is possible that item 12 “I get a minimum of 7 hours of sleep on most nights” was excluded possibly due to the characteristic sleep changes of pregnancy according to the trimester of pregnancy. By excluding this item, the scale may focus on behaviors that are directly related to a healthy lifestyle during pregnancy and not significantly influenced by the physiological changes experienced during pregnancy.

Our final solution for the EFA indicated a structure of the 2 most appropriate subscales. The items related to diet, physical activity, and mental health were distributed across both subscales. Principal component analysis aims to identify underlying patterns of relationships between items. It attempts to group items that have higher correlations with each other, suggesting that they measure similar underlying constructs.^37^ Subscale 1 includes items on social interaction, diet choices, and goal-setting behaviors, reflecting aspects of social support, eating choices, and self-efficacy to achieve goals. Subscale 2 includes items related to dietary intake, physical activity, and stress management strategies, focusing on specific health behaviors, and stress management mechanisms that contribute to general well-being during pregnancy.

Thus, some items related to diet had significant correlations with items from both subscales, indicating that they contribute to multiple dimensions of the healthy lifestyle behaviors being measured. Consider item 8 “I choose water as a beverage instead of a sugared drink,” which had a high mean score on subscale 1. This item reflects a healthy food choice by reducing the consumption of sugary drinks, like item 7. However, it is possible that other items related to diet, such as item 1 “I eat 5 servings of fruits and vegetables daily,” may also contribute to other dimensions of healthy lifestyle behaviors covered by subscale 2.

Similarly, some items related to physical activity may have shown correlations with items in both subscales. This could be due to the nature of healthy lifestyle behaviors, where certain physical activities may have an impact on multiple aspects of health, such as mental well-being. Items 2 and 3, while assessing engagement in behaviors related to physical activity, also have implications for mental health. The regular practice of physical activity during pregnancy has positive effects on mental well-being, reducing stress, anxiety, and depression symptoms.^48^ In addition, mental health-related items are distributed throughout both subscales due to their impact on healthy lifestyle behaviors, as they may influence pregnant women’s food choices and their motivation for physical activity.^49^

Conceptually, we recognized that the 2 subscales may seem similar, as both assess constructs related to healthy lifestyle behaviors. Consequently, we explored a single-factor solution during the factor analysis process. However, we found that the variance explained was significantly reduced (31.83%). This suggests that a single factor could not adequately capture the complexity of the underlying constructs measured by the scale. To resolve this limitation, we consider that future studies are necessary. To gain a deeper and more refined understanding of the dimensions underlying the healthy lifestyle measures, future studies could use confirmatory factor analysis to assess the extent to which the proposed 2-factor model fits the data or consider whether modifications to the items could improve the discriminant validity of the subscales. Thus, the distribution of items related to diet, physical activity, and mental health reflects the complex nature of healthy lifestyle behaviors. Going forward, if researchers decide to adapt the HLBES to other populations, the initial response may need to be reviewed.

The total score of the HLBES showed excellent concurrent validity with the Healthy Lifestyle Beliefs Scale, indicating a positive and significant correlation (*r* = 0.65, *P* < .01). This result is consistent with previous studies.^27,29,34^ In sum, the results of this study point to the complexity of healthy lifestyle behaviors, such as diet, physical activity, and mental health. They are not isolated elements but interconnected and can mutually reinforce each other.

### Limitations

This study has some limitations that should be considered. The HLBES is based on self-report. However, social desirability bias was minimized since the participants filled out the questionnaire anonymously. There may be potential features of healthy lifestyle behavior that may not have been captured because the scale was developed for a different population. The reason for spreading items related to diet, physical activity, and mental health across both subscales should be investigated and discussed in future study results to ensure clarity and accurate interpretation.

## Conclusions

The findings of this first study on the validity and reliability of the Portuguese version of the HLBES are a starting point. However, to establish the instrument as a valuable and widely applicable tool for measuring healthy lifestyle be-haviors in Portuguese pregnant women, further research is necessary to ensure its robustness, and generalizability. Despite the limitations of the study, these findings sugge-st that HLBES is an acceptable instrument to screen for healthy lifestyle behaviors in pregnant women and measure the effects of interventions to promote a healthy lifestyle, with implications for clinical practice and research. Future studies could focus on analyzing the sensitivity of the instrument to detect changes in healthy lifestyle behaviors across time, exploring its applicability in diverse populations and contexts, and investigating its predictive validity for perinatal outcomes. In parallel, longitudinal studies could elucidate the long-term impact of healthy lifestyle behaviors during pregnancy on maternal and child health outcomes. In addition, implementation of evidence-based intervention and cross-cultural comparative studies can be conducted employing this scale.
